# Health-related quality of life, emotional disturbances, physical functionality, perceived control, and their associations among stroke survivors: A cross-sectional study

**DOI:** 10.1371/journal.pone.0333386

**Published:** 2025-10-13

**Authors:** Shazli Ezzat Ghazali, Nor Azlin Mohd Nordin, Deepak Thazhakkattu Vasu

**Affiliations:** 1 Center for Rehabilitation and Special Needs Studies, Faculty of Health Sciences, Universiti Kebangsaan Malaysia, Kuala Lumpur, Malaysia; 2 Department of Physiotherapy, M. Kandiah Faculty of Medicine and Health Sciences, Universiti Tunku Abdul Rahman, Kajang, Selangor, Malaysia; 3 Centre for Applied Psychology and Cognitive Neuroscience, Universiti Tunku Abdul Rahman, Kajang, Selangor, Malaysia; University of Toronto, CANADA

## Abstract

**Background:**

The reintegration of stroke survivors into society is significantly and independently influenced by the survivors’ physical disabilities and emotional disturbances. In this study, we examined the relationships between stroke survivors’ emotional disturbances, physical functioning, perceived control, and health-related quality of life (QOL) during the early recovery phase, aiming to determine the predictive value of these variables for QOL outcomes.

**Methods:**

This cross-sectional study involved 66 acute stroke patients attending the outpatient rehabilitation unit of a teaching hospital in Kuala Lumpur, Malaysia. The variables of interest we measures using the Hospital Anxiety and Depression Scale (HADS) for anxiety and depression, the Euroqol-5-dimensions-5-levels (EQ-5D-5L) for QOL, the Modified Barthel Index (MBI) for physical functioning, and the Recovery Locus of Control (RLOC) scale for perceived control. Pearson’s correlation and regression analyses were performed to determine the ability of the variables to predict stroke survivors’ QOL.

**Results:**

Most of the patients were between 50 and 59 years old and were in the early stages of poststroke recovery. Although no significant gender differences were found in overall HADS scores (7.69 vs. 7.52), emotional disturbances, particularly anxiety disorders, were more prevalent among males, with 57% reporting symptoms compared to 48% of females. Health-related QOL was significantly correlated with physical functioning (*r* = −.439, *p < .001*), anxiety (*r* = .292, *p < .001*), and internal locus of control (*r* = −.224, *p < .001*). The study also revealed that QOL could be predicted by HADS (F = 4.03, *p < .001*), RLOC (F = 2.86, *p < .001*), and MBI (F = 7.46, *p < .001*) scores.

**Conclusion:**

In addition to physical disabilities, perceived control and emotional disturbances had a substantial influence on stroke survivors’ QOL outcomes. Addressing these psychosocial and behavioural factors within treatment plans is crucial for improving stroke survivors’ poststroke recovery and enhancing their QOL.

## Introduction

Recovery from stroke is primarily influenced by stroke survivors’ degrees of impairment, functional dependence, and emotional disturbance. These complex, dynamic, and multifaceted difficulties require special attention and management, and the success of stroke rehabilitation determines when individuals overcome the challenges they encounter and develop the capability to live in the community [1]. Physical disabilities and emotional disturbances play significant independent roles in stroke survivors’ reintegration into society. In addition to physical disabilities, most stroke survivors experience negative emotions, which may worsen their existing conditions, and profound, extensive changes in their diets, lifestyles, and family and social support may lead to diminished self-esteem, poor coping strategies, and cognitive impairments, in turn prompting the emergence of psychiatric disorders in the immediate poststroke phase. Unfortunately, these problems are frequently overlooked by health professionals [1], and many of these impairments and restrictions are significantly associated with longer durations of rehabilitation, higher risks of secondary stroke, and a greater cost burden on healthcare systems. Emotional disturbances further increase the burden on patients, their family members, caregivers, and health professionals, doubling the cardiovascular disease and mortality risk of caregivers, threatening their well-being, and resulting in poor-quality care and additional rehabilitation costs [2]. All of these factors eventually decrease stroke survivors’ recovery rates by influencing the factors that affect the motivation to recover, such as locus of control, self-efficacy, self-esteem, and social support, meaning that they lose confidence in their capabilities and ability to cope with difficult situations [1,3].

Mental and psychosocial disabilities are increasingly recognised as a serious health concern for stroke survivors returning to the community from acute care settings, potentially leading them to avoid participation in physical activities during the early rehabilitation phase [4].

Many of these impairments and restrictions are significantly associated with longer durations of rehabilitation, higher risks of secondary stroke, and a greater cost burden on healthcare systems and society in general. Therefore, it is imperative that the rehabilitation of stroke patients should enable stroke survivors to cope with their existing disabilities and increase their motivation to recover [5].

Emotional disturbances are a barrier to physical activity because they negatively influence adherence to exercise regimes, cause significant delays in functional recovery, and negatively affect quality of life (QOL), thereby increasing the risk of stroke recurrence due to low physical activity [6,7].

Physiotherapists play a vital role in the poststroke recovery phase as preventers, promoters, and rehabilitators by helping stroke survivors reengage in physical activities [8,9]. Intensive physical rehabilitation, as well as the proper identification and modification of these impairments, facilitate better rehabilitation and adherence to prescribed exercise programmes [8].

In this study, we explored the associations between emotional disturbances (anxiety and depression), physical functioning, perceived control, and health-related QOL among stroke survivors in the early recovery phase, aiming to assess the ability of these variables to predict QOL outcomes. The primary research question was: How do emotional disturbances, physical functioning, and perceived control impact stroke survivors’ QOL during the early recovery phase?

## Methods

### Design, setting, and sample

This cross-sectional study was based on nonprobability purposive sampling, with participants selected according to a list of inclusion and exclusion criteria. The data were collected from 66 acute stroke patients attending the physiotherapy outpatient rehabilitation centre at Hospital Universiti Kebangsaan Malaysia in Malaysia. The sample size was estimated using GPower software, based on a mixed-model analysis of variance (ANOVA) test with an effect size of 0.34 [8], a significance level of p < .05, and a study power of 80%. Cerebrovascular patients of both genders who were clinically confirmed by a neurologist were included in the study if they were within the age range of 30–75 years and had suffered a first-ever stroke within two weeks to one year after the onset of stroke. Exclusion criteria included an inability to give informed consent, having severe cognitive impairment (indicated by Montreal Cognitive Assessment (MoCA), with a cut-off value of ≥ 26), and having a comorbid psychiatric disorder other than an affective disorder (such as psychotic disorder or dementia). The Research Ethics Committee of Universiti Kebangsaan Malaysia granted ethical approval for the study (ref. UKM1.21.3/244/NN-2018–164).

### Variables and instruments

Anxiety and depression were measured using the Hospital Anxiety and Depression Scale (HADS)—a self-report questionnaire that has been confirmed as a reliable and valid instrument for identifying the presence of depression and anxiety in outpatients [10]. The recommended cut-off score for the HADS is ≥ 8 [11], and the scores classify individuals as usual (0–7), borderline (8–10), and caseness (≥ 11) for both the anxiety and depression subscales [12]. The tool has a Cronbach’s alpha (α) of 0.88 for the anxiety and 0.82 for the depression subscale.

The Euroqol-5-dimensions-5-levels (EQ-5D-5L) questionnaire is a standardised instrument for measuring generic health states. The descriptive system comprises the five dimensions of mobility, self-care, usual activities, pain/discomfort, and anxiety/depression, and each dimension comprises five levels of response: no problems, slight problems, moderate problems, severe problems, and extreme problems. Each respondent is asked to indicate their health state by checking the box next to the most appropriate statement for each of the five dimensions, which results in a one-digit number for the level selected for that dimension. The digits for the five dimensions are combined into a five-digit number that describes the respondent’s health state. All test–retest scores were reliable, with an intraclass correlation range of 0.67–0.81 [13]. In addition to the descriptive system, the EQ-5D-5L includes a visual analogue scale (VAS), on which respondents rate their overall health from 0 (the worst imaginable health) to 100 (the best imaginable health). Together, the health utility index derived from the descriptive system and the VAS score offer complementary perspectives, providing both objective and subjective assessments of health-related quality of life.

The Modified Barthel Index (MBI) was used to assess physical functioning. This scale assigns a score for the level of disability from 0 to 20 in one-point increments. It has demonstrated high test–retest reliability [14] and inter-rater reliability [15]. The recommended cutoff scores are as follows: no physical disability (20), mild impairments (15–19), moderately disabled (10–14), severely disabled (5–9), and very severely disabled (0–4).

The Recovery Locus of Control (RLOC) scale, developed by Partridge and Johnston [16], measures a person’s perceived control and individual beliefs about their health status. The scale consists of five statements designed to measure external beliefs (statements 2, 4, 6, and 8) and internal beliefs (statements 1, 3, 5, 7, and 9), with five answer options as follows: strongly agree, agree, neutral, disagree, and strongly disagree. For odd numbered questions, strongly agree is assigned five marks, and strongly disagree is assigned one mark. For even numbered questions, strongly agree is assigned one mark, and strongly disagree is assigned five marks. The total score for this questionnaire is 45, and the possible outcome range is 9–45. A higher score indicates a better locus of control. The α value for the RLOC scale is.90, and the construct validity ranges for internal and external beliefs are.77–.87 and.62–.74, respectively [17].

### Data collection procedure

Eligible respondents were briefed by the researchers about the purpose, content, and potential benefits of the study; the time required to complete the questionnaire; and their right to withdraw from the study at any time. Written consent was obtained from all participants before the study commenced. Sociodemographic data, including each patient’s age, gender, religion, marital status, and educational and clinical information, were collected, followed by administration of the HADS, MBI, EQ5D5L, and RLOC questionnaires. The data were collected from 12 December 2018–20 September 2019.

### Statistical methods

A normality test was performed following data cleaning, and the result (*p* > .05) indicated a need to use parametric statistics. Sociodemographic data were analysed using descriptive statistics. A Pearson’s correlation analysis was then performed to examine the associations between the variables, and a regression analysis was conducted to determine the ability of the variables to predict stroke survivors’ QOL.

## Results

### Sample characteristics

A total of 70 participants were screened for eligibility, and 4 were excluded from the study because they did not meet the inclusion criteria, which left 66 respondents who participated in the study (35 (53%) male, and 31 (47%) female). Most of the respondents were in the 40–49 age group (49%), 92% were Malay, 73% had primary education, and the right sides of all the participants were affected. Tables 1 and 2 present the sociodemographic and clinical profiles of the participants, as well as the mean scores of the outcome variables.

**Table 1:** Sociodemographic and clinical profiles of the participants.

Gender	n	%
Male	35	53
Female	31	47
Age (years)		
30–39	9	14
40–49	32	49
50–59	18	27
60–69	6	9
70 and above	1	1
Level of education		
SPM	48	73
Diploma	18	27
Race		
Malay	61	92
Chinese	4	6
Indian	1	2
Poststroke duration (months)		
1–6	54	82
7–12	12	18
Impaired side		
Right	66	100

**Table 2:** Participants’ EQ5D5L, HADS, RLOC, and MBI scores.

Variable	Mean (SD) Score
EQ5D5L (mobility)	2.89 (1.040)
EQ5D5L (self-care)	2.44 (.879)
EQ5D5L (usual activities)	2.55 (1.098)
EQ5D5L (pain/discomfort)	2.06 (.990)
EQ5D5L (anxiety/depression)	2.32 (1.069)
EQ5D5L (all items)	31504.59 (10885.713)
Health Utility Index	0.45989 (0.159120)
EQ5D VAS Scale	65.67 (14.543)
HADS (anxiety)	7.61 (2.359)
HADS (depression)	7.77 (2.04)
RLOC	30.86(3.42)
MBI	16.89 (2.112)

### Distribution of scores across outcome measures

#### HADS Anxiety (HADS-A).

A total of 31 participants (male = 15, female = 16) fell into the normal category, 30 (male = 16, female = 14) fell into the borderline category, and 5 (male = 4, female = 1) fell into the abnormal category for the HADS-A subscale. Table 3 summarises the baseline HADS-A scores. Male participants had a mean score of 7.69 (SD 2.589), and female participants had a mean score of 7.52 (SD 2.096). Most male participants had borderline abnormal anxiety (45%); however, 51% of the female participants fell into the normal category for anxiety.

**Table 3:** Distribution of participants’ HADS-A scores.

Gender mean (SD)	HADS-A	Total N (%)
Male 7.69 (2.60)	Normal	15 (42.90)
	Borderline abnormal	16 (45.70)
	Abnormal	4 (11.40)
	Total	35
Female 7.52 (2.09)	Normal	16 (51.60)
	Borderline abnormal	14 (45.20)
	Abnormal	1 (3.20)
	Total	31
Total	Normal	31 (47.00)
	Borderline abnormal	30 (45.50)
	Abnormal	5 (7.60)

#### HADS Depression (HADS-D).

A total of 25 participants (male = 13, female = 12) fell into the normal category, 35 (male = 18, female = 17) fell into the borderline category, and 6 (male = 4, female = 2) fell into the abnormal category for the HADS-D subscale. Male participants had a mean score of 7.82, with a standard deviation of 2.33, and females had a mean score of 7.70, with a standard deviation of 1.69. Higher percentages of patients had borderline abnormal depression (51% and 54% for males and females, respectively) (Table 4).

**Table 4:** Summarises the participants’ HADS-D scores.

Gender mean (SD)	HADS-D	Total N (%)
Male 7.82 (2.33)	Normal	13 (37.10)
	Borderline abnormal	18 (51.40)
	Abnormal	4 (11.40)
	Total	35
Female 7.71 (1.69)	Normal	12 (38.70)
	Borderline abnormal	17 (54.80)
	Abnormal	2 (6.50)
	Total	31
Total	Normal	25 (37.90)
	Borderline abnormal	35 (53)
	Abnormal	6 (9.10)

#### MBI.

Table 5 summarises the MBI baseline characteristics; most of the participants—26 male (74.3%) and 24 female (77.4%)—reported mild disability.

**Table 5:** MBI characteristics.

Gender	MBI	n (%)
Male	Moderately disabled	4 (11.4)
	Mildly disabled	26 (74.3)
	Independent	5 (14.3)
Female	Moderately disabled	1 (3.2)
	Mildly disabled	24 (77.4)
	Independent	6 (19.4)
TOTAL	Moderately disabled	5 (7.58)
	Mildly disabled	50 (75.76)
	Independent	10 (15.15)

#### RLOC.

Table 6 summarises the baseline RLOC scores. A mean score of 30.86 with a standard deviation of 3.42 (range 22–40) was obtained, and male participants had relatively high total RLOC scores.

**Table 6:** Baseline RLOC characteristics across genders.

Gender (N)	Mean (SD)	Min	Max
Male (35)	31.40 (3.84)	24	40
Female (31)	30.25 (2.81)	22	35
Total (66)	30.86 (3.42)	22	40

### Correlations between demographic and clinical factors and EQ5D5L, HADS, MBI, and RLOC scores

Table 7 shows Pearson’s correlation values for all the demographic and clinical variables. Poststroke duration was correlated with depression, which explains why a long poststroke duration can lead to emotional disturbances. Health-related QOL was significantly correlated with physical functioning (**r* *= −.439, **p* *= .000), anxiety (*r* = .292, **p* *= .001) and internal locus of control beliefs (*r* = −.224, **p* *= .014).

**Table 7:** Correlations between demographic and clinical factors and EQ5D5L, HADS, MBI, and RLOC scores.

		1	2	3	4	5	6	7	8	9	10	11	12	13	14
1	Gender														
2	Age														
3	Level of education														
4	Marital status	.304^*^ ^(p = .014)^													
5	Poststroke duration										.189* _(p = .045)_				
6	EQ5D5L							−.670** _(*p* = .000)_	−.403** (_*p* = .000)_	−.439** _(*p* = .000)_		.292** _(*p* = .001)_	−.224* _(*p* = .014)_		−.241** _(*p* = .006)_
7	Utility Index								.441** _(*p* = .000)_	.391** (_*p* = .000)_		−.296** _(*p* = .001)_	.206* _(*p* = .023)_		.208* _(*p* = .018)_
8	EQ5D VAS									.291** _(*p* = .002)_		−.205* _(*p* = .029)_			
9	MBI										−.207* _(*p* = .031)_	−.340** _(*p* = .000)_	.206* _(*p* = .033)_	.225* _(*p* = .020)_	.319** _(*p* = .001)_
10	HADS-D					.189*						.775** _(*p* = .000)_			
11	HADS-A												−.234* _(*p* = .015)_		−.259** _(*p* = .006)_
12	Internal RLOC														.432** _(*p* = .000)_
13	External RLOC	−.264^*^ _(*p* = .016)_													.635** _(*p* = .000)_
14	Total RLOC														

**p < .01, *p < .05; Pearson’s correlations.

### Influence of RLOC and emotional disturbances on the prediction of physical functioning

Table 8 shows the hierarchical regression results for the influence of post-stroke duration, RLOC and emotional disturbances on the prediction of physical functioning. The results showed that the post-stroke duration contributed 1% to physical functioning. The emotional disturbances and RLOC were found to significantly predict physical functioning. Emotional disturbances accounted for an additional 13.4% of the variance in physical functioning, with the model being significant (F(3,62)=4.35,p < .05). The RLOC scores (both internal and external) further added to the prediction, contributing a significant 16.7% of the variance, statistically significant (F(5,60)=4.35,p < .05), indicating a strong overall predictive relationship.

**Table 8:** Hierarchical regression results for emotional disturbances (anxiety/depression) and RLOC domains.

Variables entered	B value	95% CI	*p* value	R^2^	R^2^Δ
Step 1					
Poststroke duration	−.077	−0.27, 0.11	.43	.010*	
Step 2					
Poststroke duration	−.036	−0.22, 0.15	.699	.174	.134
Poststroke anxiety	−.56	−1.02, −0.10	.017*		
Poststroke depression	.27	−0.25, 0.81	.304		
Step 2					
Poststroke duration	−.03	−0.22, 0.14	.666	.231	.167
Poststroke anxiety	−.40	−0.91, 0.10	.115		
Poststroke depression	.18	−0.38, 0.74	.519		
IRLOC	.150	−0.09, 0.4	.233		
ERLOC	.17	−0.01, 0.36	.071		

(*p < .05).

### Influence of RLOC, MBI and emotional disturbances on the prediction of QOL

Table 9 presents the results of a hierarchical regression analysis showing how emotional disturbances, RLOC and MBI affect the prediction of QOL. The analysis revealed that emotional disturbances (anxiety and depression scores) significantly accounted for 12.3% of the variance in QOL, which explained a significant proportion of the variance (F (2,62) = 4.03, p <.05). The results showed that poststroke duration contributed 5.7% of the QOL score, while emotional disturbances contributed approximately 12.3% of the score, which explained a significant proportion of the variance (F (2,62) = 4.03, p <.05). The results also showed that 12.5% of QOL could be predicted by the RLOC score (F (3,60) = 2.86, p <.05), with physical functioning contributing approximately 37.4% of the QOL score (F (4,60) = 7.46, p <.05).

**Table 9:** Hierarchical regression results for poststroke duration, emotional disturbances (anxiety and depression), the RLOC domains and MBI.

Variables entered	B value	95% CI	*P* value	R^2.^ value	R^2^Δ
Step 1					
Poststroke duration	.014	0.000,0.028	.054	.057	.42 (*p* = .054)
Step 2					
Poststroke duration	.017	0.002,0.031	.022	.163	0(*p* = .011)
HADS-A	−.034	−0.069,0.001	.056		
HADS-D	.017	−0.024,0.057	.419		
Step 3					
Poststroke duration	.017	0.003,0.032	.017*	.193	.125 (*p *= .022)
HADS-A	−.021	−0.060,0.018	.282		
HADS-D	.005	−0.038,0.049	.805		
IRLOC	.014	−0.005,0.033	.146		
ERLOC	.000	−0.014,0.015	.957		
Step 4					
Poststroke duration	−.326	.007, 0.031	.002*	.423	.374 (*p *= .000)
HADS-A	−.004	−0.038, 0.030	.804		
HADS-D	−.002	−0.039, 0.035	.902		
IRLOC	.008	−0.009, 0.024	.343		
ERLOC	−.007	−0.020, 0.006	.280		
MBI	.042	0.025, 0.059	.000*		

(*p* < .05).

## Discussion

Sixty-six individuals who had experienced a first-time stroke participated in this study, most of whom were aged 50–59 years and in the early stages of poststroke recovery. Poststroke anxiety and depression occurred in one-third of the stroke survivors and were the most common emotional complications in the early stroke recovery phase. In this study, there was an increasing trend of anxiety over time. The mean HADS-A score rose from a lower level in the acute phase to a higher level at nine months and beyond, indicating that anxiety levels worsened as the post-stroke duration increased. These findings align with a systematic study conducted by Campbell et al. [18].

Regarding depression, more than half of the participants were identified as having a high risk of depression. Prior studies have noted the importance of early screening for poststroke depression because it tends to be highest in the early poststroke recovery phase, as observed by Towfighi et al. [19] According to previous literature [20], gender does not predict poststroke depression; in this study, the majority of both male and female participants demonstrated symptoms of depression, which may be associated with the high prevalence of physical disabilities in the cohort.This result confirms that the development of depression is associated with more severe disability [12].

A gender comparison revealed no significant differences; however, about half of both male and female participants reported experiencing anxiety, with the slightly higher proportion among males possibly related to their greater prevalence of physical disability. This finding confirms that physical disability has a strong influence on poststroke anxiety [12].

The mean RLOC score was higher in male participants with a high percentage of physical disabilities, possibly due to the influence of the lower mean age of males than females [17].

The participants’ functional abilities were assessed in this study using the MBI, and most participants fell into the mildly disabled category. The overall mean MBI score was high, but an increasing trend was noted in the early poststroke recovery phase, possibly due to spontaneous neurological recovery. Regarding the gender variation in MBI scores, female participants had a lower mean score than male participants, possibly due to their low physical activity and poor effective coping mechanisms for dealing with health issues, as explained by Lee et al. [21] Moreover, female participants were slightly older than the male participants, and these findings align with those of Musa et al.’s study [22].

The results of the correlation analysis showed that poststroke duration was linked to depression, suggesting that the longer a person has been poststroke, the likelier they are to experience emotional difficulties. The study findings also revealed that health-related QOL was strongly connected to physical functioning in stroke patients. This suggests that individuals’ physical abilities after strokes significantly impact their overall perceptions of their health and well-being. The results emphasise the importance of focusing on physical rehabilitation and recovery for stroke survivors to improve their QOL.

The study revealed a significant correlation between health-related QOL and both anxiety and locus of control in individuals who had experienced strokes. Higher levels of anxiety can negatively affect a person’s overall perception of their health and well-being, while a person’s confidence in their control over events and life circumstances, referred to as a locus of control, can also impact their health-related QOL after a stroke. The participants with internal locus of control tended to perceive their health and well-being positively, whereas those with external locus of control had more negative perceptions. These findings emphasise the importance of addressing psychological factors, such as anxiety and locus of control, in comprehensive treatment plans for stroke patients to improve their QOL.

The levels of participation and recovery of stroke patients can be objectively estimated by measuring their social participation, return-to-work rates, and performance at work [23]. Psychological disturbances, as well as physical and sociodemographic factors, are known to predict participation restrictions [23]. Research has shown that low QOL and a poor prognosis are associated with anxiety and depression [24,25].

Anxiety and depression can present in various ways and often limit stroke survivors’ ability to recognise their limitations, set poststroke goals, learn new skills, and follow rehabilitation programmes [25]. Poststroke anxiety can also affect mental processing, mood, and alertness, leading to emotional instability, poor disability acceptance, impaired concentration, and unrealistic expectations [26], all of which can negatively impact stroke survivors’ poststroke recovery and QOL [27].

During rehabilitation, patient engagement and feedback are essential for helping stroke survivors achieve their goals. However, anxiety and depression often result in poor motivation regarding rehabilitation interventions, leading to delayed adaptation, increased burdens on healthcare providers and caregivers, and additional healthcare costs due to slow poststroke recovery [28].

During the early stages of stroke recovery, survivors may experience low acceptance of their physical disabilities and communication and cognitive impairments, leading to anxiety and depression [26]. These symptoms can further hamper recovery by affecting motivational factors such as locus of control, self-efficacy, self-esteem, and social support [29], potentially causing survivors to lose their belief in their capabilities and to struggle with difficult situations [1,3].

Research has shown that the relationship between RLOC scores and emotional disturbances can have a significant impact on the prediction of QOL in individuals who have experienced strokes [27]. Studies have shown that individuals with an internal locus of control, or a belief that they can control events and their life circumstances, tend to have more positive perceptions of their health-related QOL than those with an external locus of control [23,30]. However, poststroke anxiety and depression are considered predictors of poor QOL and prognoses [24,25].

Anxiety and depression can cause stroke survivors to have low motivation for rehabilitation, which can delay their recovery and increase healthcare and caregiver burdens [28]. In particular, psychosocial and behavioural impairments are considered a significant health concern when stroke survivors return to the community from inpatient care settings. Low disability acceptance levels during the early phase of stroke recovery, combined with physical disabilities and communication and cognitive impairments, can result in further anxiety and depression, which can negatively impact factors such as locus of control, self-efficacy, self-esteem, and social support [26,29,30]. This can lead to a loss of belief in one’s capabilities and difficulties in coping with difficult situations [1,3], in turn prompting the emergence of psychiatric disorders. Unfortunately, these poststroke problems are frequently overlooked. About 65% of stroke survivors become nonadherent to home workout regimes, and 10% do not complete their scheduled physiotherapy exercises [31]. Consequently, they may require long-term care and financial support to maintain or improve their existing functioning.

Physical exercise plays a potential role in alleviating depression and anxiety symptoms; it also prevents and reduces emotional imbalances by improving functional independence. Apart from intensive physical exercise as a primary mode of rehabilitation, stroke survivors need motivational interventions to promote their participation in rehabilitation programmes. Many international guidelines recommend that evidence-based interventions and strategies should be introduced to change existing behaviour patterns [32,36].

Embedding psychological interventions within physiotherapy sessions has proven to be beneficial in supporting patients’ recovery and well-being [31,33]. Incorporating relaxation exercises, particularly those intended to reduce emotional distress, is effective in mitigating the negative impact of strokes [34,35,37,38]. Such interventions promote general body relaxation, enhance psychosocial functioning, and increase patients’ motivation [34]. Furthermore, they are associated with improved self-perceived QOL and greater empowerment [32,35]; therefore, early screening for psychological disturbances should be integrated into routine stroke rehabilitation protocols.

This study has a few limitations that should be acknowledged. Firstly, the relatively small sample size and single-centre design may limit the generalizability of the findings. Secondly, the cross-sectional nature of the study restricted the ability to draw causal inferences between the variables examined. Future researchers should consider recruiting larger, more diverse samples across multiple sites and employing longitudinal designs to better understand the temporal dynamics and relationships among the studied variables.

In conclusion, both locus of control and emotional disturbances play a significant role in predicting QOL in individuals who have experienced strokes. Addressing these psychological factors should be a crucial component of comprehensive treatment plans to improve QOL for stroke survivors.

## Conclusion

In conclusion, anxiety and depression are common sequelae of stroke, with symptoms ranging from mild anxiety to severe depression. These psychosocial and behavioural impairments have a significant impact on stroke survivors’ recovery, affecting their ability to recognise their limitations, set poststroke goals, and participate in rehabilitation programmes. Low levels of acceptance regarding physical disabilities and communication and cognitive impairments, and the influence of factors such as locus of control, self-efficacy, self-esteem, and social support, can lead to low motivation and emotional instability, in turn leading to poor self-motivation, slow poststroke recovery, and higher health and social care costs. Therefore, it is crucial to consider these psychosocial and behavioural factors in comprehensive treatment plans for stroke patients to improve their QOL and functional recovery.
